# The quality of life of frequently vs. infrequently screened HPV vaccinated women

**DOI:** 10.1007/s11136-023-03575-y

**Published:** 2024-01-18

**Authors:** Kaisa Taavela, Tiina Eriksson, Heini Huhtala, Anne Bly, Katja Harjula, Kaisa Heikkilä, Mari Hokkanen, Mervi Nummela, Laura Kotaniemi-Talonen, Matti Lehtinen, Karolina Louvanto

**Affiliations:** 1 Faculty of Medicine and Health Technology, Tampere University, Arvo Ylpön Katu 34, 33014 Tampere, Finland; 2 Department of Obstetrics and Gynecology, Tampere University Hospital, Tampere, Finland; 3 FICAN-Mid, Tampere, Finland; 4 Faculty of Social Sciences, Tampere University, Tampere, Finland; 5 Department of Laboratory Medicine, Karolinska Institute, Stockholm, Sweden

**Keywords:** Human papillomavirus, HPV, HPV vaccination, Quality of life, Cervical cancer screening

## Abstract

**Purpose:**

Cervical lesions caused by human papillomavirus (HPV) are related to decreased quality of life (QoL) of women. Also, cervical cancer (CC) screening can cause psychological adverse effects. It has been assumed that by decreasing the HPV-related disease burden, HPV vaccinations would increase the QoL. This study compares the effect of CC screening on QoL of HPV vaccinated women in two different screening protocols.

**Methods:**

A total of 753 HPV16/18 vaccinated women were randomized to frequent (22/25/28 years of age) and infrequent (28 years of age) CC screening arms. QoL questionnaires (EQ VAS, RAND 36, amended CECA 10) were sent at the age of 28.

**Results:**

Median EQ VAS scores were 80 (Q_1_–Q_3_ 75–90) in both screening arms. Mean RAND 36 scores of frequently and infrequently screened women were 78.13/81.64 in Physical role functioning domain and, respectively, 77.93/80.18 in Pain, 69.10/69.12 in General Health, 54.67/53.61 in Energy, 83.72/85.11 in Social functioning, 69.53/69.68 in Emotional role functioning, and 68.16/69.29 in Emotional well-being domain. Among women with a self-reported history of Pap cytology abnormalities, overall mean scores of amended CECA 10 were 69.52/72.07, and among women with a self-reported history of genital warts, 60.09/66.73, respectively.

**Conclusion:**

There was no significant difference in the QoL of HPV vaccinated women between the two CC screening arms. Women were mostly satisfied with the screening experience despite the screening frequency. This information is important for the future screening program planning as we need to reach the best possible balance with screening benefits and harms.

**Trial registration number:**

NCT02149030, date of registration 29/5/2014.

## Plain English summary

Vaccinations against human papillomavirus have been expanded to many countries during recent years, and the aim is to reduce the number of cervical cancers together with cervical cancer screening programs. The quality of life of vaccinated women may be affected by cervical cancer screening as screening and related examinations are known to cause anxiety and fear of cancer. Vaccinated women are coming to the screening age and there is a great need to design new screening programs for them. This study investigated the quality of life of vaccinated women in two different screening arms, frequently and infrequently screened. We found no difference in quality of life between the two screening arms. Cervical cancer screening seems to be well accepted among women and more frequent screening did not reduce the quality of life. On the other hand, vaccinated women seem to feel safe also despite the more infrequent screening as the quality of life was good also among women in this arm. In future, it will most likely be appropriate and safe to reduce the frequency of cervical cancer screening among vaccinated women.

## Background

Human papillomaviruses (HPVs) are common DNA viruses that infect epithelial cells and cause a variety of epithelial lesions ranging from common warts to intraepithelial neoplasia and cancer [1]. 1It is widely acknowledged that approximately 80% of women will be infected with HPV during their lifetime and persistent high-risk (HR) HPV types are necessary for the development of all cervical cancer (CC) [2]. For HPV prevention, HPV vaccination has been introduced so far in more than 100 countries worldwide [3]. HPV vaccination has shown to be effective and safe [4]. In long term, together with CC screening programs, HPV vaccinations will greatly reduce HPV-related disease burden around the world [5, 6].

It is known that HPV-related diseases negatively affect the quality of life (QoL) of women in several ways [7, 8]. Diagnosed HPV infection can in worst case disrupt women’s interpersonal and sexual relationships [9]. There is also increasing amount of knowledge on psychological adverse effects of cervical lesions and associated examinations and treatments. Abnormal Pap cytology, positive HPV test result, and colposcopy with or without biopsies or treatment are known to provoke significant anxiety on women. [10–13] Most worries concern the possibility of being diagnosed with CC, but also concerns on future fertility and sexuality have been commonly reported [14, 15]. Also, short-term decrease in sexual function has been reported after loop electrosurgical excision procedure (LEEP) of cervical dysplasia [16]. Younger women present higher concerns about their future, health, and fertility [15]. This might be due to the association of cervical HPV infection, precancer, and their treatments with increased risk of premature birth [17], and in some studies also with miscarriages [18].

The increasing interest in the personal viewpoint of patients’ health has led to a demand for reliable and valid standardized questionnaires of health-related quality of life (HRQoL), and a variety of questionnaires have been developed to meet this need [19]. HRQoL is referring to patient's perceptions of the impact of disease and treatment on their physical, psychological, and social function and well-being [20].

In countries with established or emerging HPV vaccination programs and pre-existing screening, there is a great need to design the most optimal future cervical cancer screening program for HPV vaccinated women. This is currently highly crucial as the first birth cohorts of HPV vaccinated women are approaching the screening age in many countries. Among HPV vaccinated women, severe HPV-related diseases will occur more rarely compared to unvaccinated women, and a highly reduced screening program, even only once in a lifetime screening, may be enough and safe [21]. With the current study, our aim was to compare the HRQoL between frequently and infrequently screened HPV vaccinated women and provide information on CC screening related QoL to support future screening planning.

## Methods

### Participants

The current study consists of 753 HPV16/18 vaccinated women who originally participated in the community-based Finnish HPV vaccination trial as previously described [22]. Bivalent HPV vaccine was given to the participants at the age of 13–15. At the age of 22, they were re-randomized to frequent (FS) and infrequent screening (IFS) arms. All women in both arms came to the screening visits three times during the study period, at the age of 22, 25, and 28. Cervical samples were taken from all women during every visit. Women in frequent screening arm got to know their screening results after every screening visit, while women in infrequent screening arm got to know their results only after their last follow-up visit. As an exception, also infrequently screened were informed about their earlier results if they were indicative of colposcopy referral (ASC-H, HSIL, or AGC in Pap cytology). At the age of 28, a survey considering background information, opinions on screening and HRQoL were sent to participants in both screening arms after their latest CC screening results. The validated HRQoL questionnaires included EuroQol Visual Analogue Scale (EQ VAS), the Finnish version of the RAND 36-Item Health Survey (RAND 36), and amended CECA (a Spanish acronym for the Specific Questionnaire for Condylomata Acuminata, Cuestionario Específico para Condiloma Acuminado) that are described in detailed under.

### EuroQol Visual Analogue Scale (EQ VAS)

EQ VAS is a standardized, validated generic questionnaire in which the patient describes the experienced general state of health in a scale from 0 indicating worst imaginable health state to 100 indicating best imaginable health state [23].

### Finnish version of the RAND 36-Item Health Survey (RAND 36)

RAND 36 is a standardized, validated generic questionnaire which assesses health and well-being in eight different domains: Role limitations due to physical health problems, Physical functioning, Pain, General health, Energy, Social functioning, Role limitation due to emotional problems, and Emotional well-being. RAND 36 scores range from 0 indicating worst HRQoL to 100 indicating best HRQoL. Scoring is based on answers to combinations of questions. [24]

### Cuestionario Específico para Condiloma Acuminado, Spanish acronym for the Specific Questionnaire for Condylomata Acuminata (CECA 10)

CECA 10 is a disease-specific HRQoL questionnaire for patients with genital warts [25]. Earlier studies have shown that the CECA 10 questionnaire is a valid, reliable, and sensitive tool for the assessment of HRQoL. The questions refer to the past 7 days. Questionnaire comprises 10 items and 2 dimensions. The emotional dimension includes 6 items, and the sexual activity dimension includes 4 items. Each question allows an answer on a 5-option Likert scale indicating the degree of agreement: 1 = “totally agree,” 2 = “agree,” 3 = “don’t know,” 4 = “disagree,” and 5 = “totally disagree.” The global scoring range is 10–50, ranging from 6 to 30 in the emotional dimensions and from 4 to 20 in the sexual activity dimension. All dimensions are standardized for a scoring between 0 indicating worst HRQoL and 100 indicating best HRQoL. [26] In our study CECA 10 was minimally modified and therefore used for women with a self-reported history of Pap cytology abnormalities in addition to women with a self-reported history of genital warts.

### Statistical analysis

RAND 36 questionnaire was modified in our study as Physical functioning -domain was omitted from the questionnaire because it wouldn’t have been usable among the young population of this study. RAND 36 scores were calculated following the general scoring instructions of the questionnaire [24]. Scores were calculated for each domain if a woman had answered to at least half of the questions of the current domain and had answered to all questions of the domain of Pain and Social functioning. Otherwise, women were excluded from the RAND 36 analysis. In our study, amended CECA 10 questionnaire was intended only for women who reported a history of Pap cytology abnormalities or genital warts in the survey. Screening results were not verified in this context by the researchers. Emotional and sexual activity dimension as well as overall scores were calculated separately for each dimension using those that had a reported answer. Scores for each dimension were calculated only for women who had answered all questions of the dimension.

Survey included five questions concerning women’s opinion about CC screening, more precisely on whether Pap cytology sampling was a pleasant experience, whether women were sufficiently informed before Pap cytology sampling and whether they knew how to get the test results. Questions are shown in Table 2 with results. Each question allowed an answer on a 5-option Likert scale: 1 = ”absolutely true,” 2 = ”mostly true,” 3 = ”don’t know,” 4 = ”mostly not true,” and 5 = ”absolutely not true.” In questions 1–4, answers 1 and 2 (1 = “absolutely true” and 2 = “mostly true”) were combined to illustrate satisfaction on Pap cytology sampling. In question 5, answers 4 and 5 (4 = “mostly not true,” 5 = “absolutely not true”) were combined to illustrate satisfaction.

Statistical analyses were performed using Stata version 17.0. FS and IFS arms were compared using statistical tests suitable for each variable. P value ≤ 0.05 was considered statistically significant. Chi-squared or Mann–Whitney rank-sum test, and if the expected frequency in each cell was below five in at least 80% of the cells, Fischer exact test, was used to compare the background characteristics and different scores among the women in FS and IFS arms.

## Results

A total of 753 responses out of 1808 invitations (41.6%) were received, of which 370 were from the FS arm and 383 from the IFS arm. Majority of the women in both arms were from the birth cohort of 1993. Sociodemographic, as well as clinical characteristics and different sexual behaviors of the women are shown in Table 1. There was no significant difference between the two arms in most of the background characteristics. Small differences were observed in the proportions of different birth control methods used between the women in different study arms, as interrupted intercourse was more common method of pregnancy prevention among frequently screened than infrequently screened women (37.4% vs. 30.0%, *p* = *0.050*). The opposite was observed for hormonal IUD usage (21.8% vs. 28.1%, *p* = *0.035*). Also, the proportion of women who had had oral sex with the latest partner was higher among the infrequently screened women (82.8% vs. 89.2%, *p* = *0.013*) but no difference was found in the frequency of oral sex protection use habits between the arms. Women in the frequently screened arm reported more often having a history of Pap cytology abnormalities (21.2% vs. 14.0%, *p* = *0.010*). On the other hand, no difference in the reported history of genital warts was observed.

**Table 1:** Background information of the frequently and infrequently screened HPV vaccinated women at the age of 28. Statistically significant results are shown in bold^a^

	Frequently screened		Infrequently screened		*p* value
	*n* = 370		*n* = 383		
	*n*	%	*n*	%	
Year of birth					0.853
1992	66	17.8	67	17.5	
1993	231	62.4	246	64.2	
1994	73	19.7	70	18.3	
Working/studying					0.881
Working	248	69.5	261	70.2	
Studying	36	10.1	32	8.6	
Not working or studying	32	9.0	32	8.6	
Working and studying	41	11.5	47	12.6	
Study place					0.523
Higher education	43	53.8	44	53.7	
Polytechnic	22	27.5	27	32.9	
Upper secondary vocational school	10	12.5	5	6.1	
Apprenticeship	1	1.3	3	3.7	
Other	4	5.0	3	3.7	
Contraceptive method					
None	0	0	2	0.5	0.499
Combined contraceptive product (E + P) or progestin pills	337	94.1	351	93.1	0.783
Progestin implant	32	8.9	29	7.7	0.588
Condom	351	98.0	359	95.2	0.504
Pessary, spermicides	1	0.3	5	1.3	0.217
Interrupted intercourse	**134**	**37.4**	**113**	**30.0**	0.050
Emergency contraception	213	59.5	235	62.3	0.290
Hormone IUD	**78**	**21.8**	**106**	**28.1**	0.035
Copper IUD	17	4.7	17	4.5	0.918
Smoking					0.495
Never smoked	235	64.4	234	61.4	
Former smoker	72	19.7	74	19.4	
Current smoker	58	15.9	73	19.2	
Married or in a permanent relationship	285	77.2	307	80.2	0.328
Have had sexual intercourse	358	96.8	376	98.4	0.134
Age at first sexual intercourse (years)					0.487
12–14	66	18.6	59	15.8	
15–17	177	49.9	203	54.3	
18–20	85	23.9	93	24.9	
21–23	21	5.9	15	4.0	
≥ 24	6	1.7	4	1.1	
Number of lifetime sexual partners					0.717
1	40	11.3	44	11.7	
2	26	7.3	33	8.8	
3	23	6.5	22	5.9	
4	16	4.5	26	6.9	
5–9	97	27.3	96	25.6	
≥ 10	153	43.1	154	41.1	
Number of sexual acts during past year with the most recent sexual partner					0.348
0	14	4.1	15	4.2	
1–20	113	32.9	116	32.1	
21–50	109	31.8	113	31.3	
51–100	73	21.3	94	26.0	
> 100	34	9.9	23	6.4	
Number of sexual acts during past year with previous sexual partner					0.523
0	66	36.5	67	38.5	
1–20	77	42.5	65	37.4	
21–50	20	11.0	27	15.5	
51–100	13	7.2	13	7.5	
> 100	5	2.8	2	1.1	
Frequency of condom use with the most recent partner					0.072
Never	177	51.2	204	56.2	
Half of the times	35	10.1	50	13.8	
Every time	78	22.5	67	18.5	
Other	56	16.2	42	11.6	
Practicing oral sex with the most recent partner	**293**	**82.8**	**323**	**89.2**	0.013
Using oral sex protection with the most recent partner	10	3.4	9	2.8	0.653
Self-reported history of Pap cytology abnormalities	**76**	**21.2**	**53**	**14.0**	0.010
Self-reported history of genital warts	31	9.0	33	9.1	0.943

*E* estrogen, *P* progestin

^a^*p* value ≤ 0.05

EQ VAS scores that evaluated the experienced general state of health did not show a significant difference between FS and IFS arm. In a scale from 0 to 100, median EQ VAS was 80 (Q_1_–Q_3_ 75–90) in both arms (data not shown).

RAND 36 results are shown in Table 2. There was no significant difference between the two arms in any domain. However, a trend of lower scores was seen in six of seven domains among frequently screened women compared to infrequently screened. Energy domain was an exception to this trend.

**Table 2:** RAND 36-Item Health Survey ^a^ scores of frequently and infrequently screened HPV vaccinated women at the age of 28

	Frequently screened		Infrequently screened		*p* value
	*n* = 370		*n* = 383		
	Mean	SD	Mean	SD	
Physical role functioning	78.13	32.23	81.64	30.47	0.128
Pain	77.93	20.78	80.18	19.48	0.128
General health	69.10	19.00	69.12	18.65	0.989
Energy	54.67	19.46	53.61	20.43	0.471
Social functioning	83.72	19.67	85.11	20.00	0.344
Emotional role functioning	69.53	38.47	69.68	36.47	0.956
Emotional well-being	68.16	18.17	69.29	17.49	0.385

RAND 36 scores range from 0 (worst HRQoL) to 100 (the best HRQoL)

*SD* standard deviation

*p* value ≤ 0.05 was considered statistically significant

^a^Physical functioning domain omitted

Amended CECA 10 scores of women with a self-reported history of Pap cytology abnormalities or genital warts are shown in Table 3. A total of 106 (14.1%) women had answered to the amended CECA 10 questionnaire which was intended for women with a history of Pap cytology abnormalities. There was no significant difference in amended CECA 10 scores between the two arms. However, a trend of lower scores was seen in all amended CECA 10 dimensions among frequently screened women compared to infrequently screened. In addition, a total of 55 (7.3%) had answered to the amended CECA 10 questionnaire intended for women with a history of genital warts. There was no significant difference in amended CECA 10 scores between the arms. Again, a trend of lower scores among frequently screened women was observed in emotional and overall scores, though.

**Table 3:** Amended CECA 10 scores of frequently and infrequently screened HPV vaccinated women who reported a history of Pap cytology abnormalities or genital warts

	Frequently screened		Infrequently screened		*p* value
	Mean	SD	Mean	SD	
Pap cytology abnormalities (n (FS/IFS) = 64/42)					
Emotional dimension	63.22	27.59	64.23	22.13	0.877
Sexual dimension	80.00	25.39	84.23	17.74	0.822
Overall	69.52	24.09	72.07	17.56	0.792
Genital warts (n (FS/IFS) = 29/26)					
Emotional dimension	55.32	30.62	65.87	24.92	0.190
Sexual dimension	68.29	30.32	68.03	28.63	0.885
Overall	60.09	28.16	66.73	25.01	0.359

CECA 10 scores range from 0 (the worst HRQoL) to 100 (the best HRQoL)

*CECA* Spanish acronym of specific questionnaire for condylomata acuminate, *FS* frequently screened, *IFS* infrequently screened, *SD* standard deviation

*p* value ≤ 0.05 was considered statistically significant

Five questions in the survey concerned the women’s opinion about CC screening. Results are shown in Table 4. There was no significant difference between the two arms in the proportion of women who were satisfied with the Pap cytology sampling. Depending on the specific question, proportions of satisfied women were 73.4–97.5% and 74.3–96.8% in FS and IFS arms, respectively. The survey included one question about overall satisfaction in the current life situation at the age of 28. In a scale from 0 to 10, median score in both arms was 8 (Q_1_–Q_3_ 7–9) (data not shown.)

**Table 4:** Proportions of frequently and infrequently screened HPV vaccinated women who were satisfied with the Pap cytology sampling experience

	Frequently screened	Infrequently screened	*p* value
	*n* = 370	*n* = 383	
	%	%	
Pap cytology sampling was considered as a pleasant experience	73.4	74.3	0.803
Enough information about sampling was given before the visit	94.0	93.2	0.658
Women knew how to prepare for sampling before the visit	93.2	93.7	0.770
Enough advice and guidance were received regarding the sampling situation	97.5	96.8	0.660
Women were aware of how and when she would get the Pap cytology test result	83.7	86.4	0.307

*p* value ≤ 0.05 was considered statistically significant

## Discussion

These results are, to our knowledge, the first to evaluate the effect of screening frequency on the QoL of HPV vaccinated women. It has been expected that HPV vaccinations would improve HRQoL by reducing incidence of HPV-related lesions. However, earlier studies have shown that CC screening lowers the HRQoL in long term.

In our study, the frequency of CC screening did not significantly affect the HRQoL of young HPV vaccinated women. At first, this seems quite unexpected, as in previous studies among unvaccinated women, the history of genital warts or cytological abnormalities, the conditions commonly related to frequent smear sampling and healthcare visits have been associated with reduced QoL [7, 8, 27–29]. Especially anogenital warts, caused by low-risk HPV types, have shown to cause psychological distress by making patients feel more ashamed of themselves and less attractive. In addition, diagnosis and treatment of genital warts have shown to trigger high level of anxiety, anger, and depression, and approximately, two-thirds of patients have made lifestyle changes especially regarding their sexual relationships [9]. Moreover, women with anogenital warts are shown to have fears about sexual transmission which affects their self-image and life control perception [7, 30], and sexual enjoyment and activity are negatively affected [31]. However, no difference has been yet reported between HPV vaccinated and unvaccinated women [8, 32]. In our study, neither the rates of self-reported anogenital warts, nor the number of actual cervical screening samplings differed between study arms, which is one of the probable explanations behind the similar HRQoL scores.

Earlier studies have suggested that cervical lesions as well as associated examinations and treatments cause psychological adverse effects [10–12]. During the current study, participating women met a health care professional regularly and had therefore the opportunity to ask questions and receive advice. This may have lessened their concerns about HPV-related diseases and made them feel safe and taken care. Thus, also frequently screened women may have stayed less anxious when waiting for and getting the screening results. However, a trend of lower scores was seen in amended CECA 10 and RAND 36 scores among frequently screened women compared to infrequently screened but the difference was not statistically significant. These results support the hypothesis that women might be more distressed of repeated screening. It is possible, that if the length of follow-up were longer and the difference between study arms in the number of screening visits were wider, also differences between HRQoL indicators would have been observed. Further, it is worth noticing that all the participating women had accepted as adolescents to participate HPV vaccination trial and might have more positive attitude toward health care interventions as general population, which may have diluted the difference in HRQoL between the arms.

Disease-specific questionnaires that only contain items specifically designed for a particular condition are more likely to be relevant and sensitive to patients in areas that clinicians may wish to monitor [25]. The CECA 10 questionnaire has been developed and validated for genital warts and not for cytological abnormalities. However, earlier studies have used it also in this patient group and it has been suggested relevant [8]. In our study, the survey was sent after the women had received their results from the latest screening. There was no significant difference in amended CECA 10 scores between the two arms among women with a self-reported history of genital warts or Pap cytology abnormalities. The history of these anogenital or cervical changes was based on the women’s answers in the survey. Screening results were not verified in this context by the researchers. Earlier studies have shown that HPV-related QoL is worse at initial diagnosis than in later assessment [33]. This is understandable as the HPV infection commonly disappears within 1–2 years. This may influence our results since patients were already at 28 years of age and some earlier Pap cytology abnormalities and genital warts may have resolved before answering the survey.

Liddon et al. studied HPV vaccination uptake and sexual behavior among adolescent and young women. In their study, HPV vaccination was not associated with being sexually active or with number of sex partners. Among sexually active adolescents, vaccinated girls were more likely to always use a condom. [34] Studies on the effect of contraceptives on cervical dysplasia have been controversial [35, 36]. It is not known how this might influence women’s contraceptive use. In our study, sexual activity was equal among vaccinated women in both screening arms considering the number of sexual partners and the number of sexual sessions during last year. In our vaccinated population, there was no difference in the frequency of condom use between the frequently and infrequently screened women. Interrupted intercourse was more common pregnancy control method in frequently screened arm. Women in the infrequently screened arm were more likely to use hormonal IUD. There was no significant difference in other hormonal contraceptive product use between the arms.

Our study has some limitations. The response rate was relatively low, which is typical for questionnaire studies, and selection bias may exist. It is possible that responded women had more motivation to take part to the study. However, the response rate was very similar in both arms, and therefore, we estimate that the possible selection bias is equal in both arms and did not impact much these results. The follow-up time was six years and CC screening samples were taken in three-year intervals. Short timeframes between the screening visits might have diluted the differences in the QoL between the study arms. More studies on how CC screening affects the QoL among HPV vaccinated women would be valuable to achieve more in-depth information on women´s view on the screening experience. Longer follow-up time and including a reference arm of unvaccinated women with an additional depression scale questionnaire could give a more comprehensive understanding on the QoL and CC screening.

The current CC screening programs are in verge of changes as the first HPV vaccinated women are currently approaching the CC screening age in many countries. CC screening programs will need to be adjusted for HPV vaccinated women to be able to provide them the best possible screening tests and schedule. When planning future CC screening, it is important also to consider women’s opinion about screening. In our study, there was no significant difference between the arms in the proportion of women who were satisfied with the Pap cytology sampling experience; both the frequently and infrequently screened women were mostly satisfied with screening. Thus, CC screening seems to be well accepted among HPV vaccinated women from the individual’s point of view. HPV vaccinations reduce the prevalence of HR-HPV infections and cervical cancer, and vaccinated women may therefore feel safer also in infrequent screening program. This information is important for the future screening program planning for HPV vaccinated women. A highly reduced screening program is likely to be safe due to the lower prevalence of HPV-related diseases. We need to reach the best possible balance with screening benefits and harms. Cost-effectiveness analysis of screening must also be considered. More infrequent CC screening of HPV vaccinated women in future will most likely be cost-effective and safe without a negative effect to the QoL.

## Data Availability

Individual participant data that underlie the results reported in this article, after de-identification, will be available for researchers who provide a methodologically sound proposal with achievable aims. Proposals should be directed via e-mail to the corresponding author (KL); data requestors will need to sign a data access agreement.
